# Systematic Assessment of Seven Solvent and Solid-Phase Extraction Methods for Metabolomics Analysis of Human Plasma by LC-MS

**DOI:** 10.1038/srep38885

**Published:** 2016-12-21

**Authors:** Dmitri G. Sitnikov, Cian S. Monnin, Dajana Vuckovic

**Affiliations:** 1 Department of Chemistry and Biochemistry, Concordia University, 7141 Sherbrooke St. W., Montréal, Québec, H4B 1R6, Canada; 2 PERFORM Centre, Concordia University, 7141 Sherbrooke St. W., Montréal, Québec, H4B 1R6, Canada

## Abstract

The comparison of extraction methods for global metabolomics is usually executed in biofluids only and focuses on metabolite coverage and method repeatability. This limits our detailed understanding of extraction parameters such as recovery and matrix effects and prevents side-by-side comparison of different sample preparation strategies. To address this gap in knowledge, seven solvent-based and solid-phase extraction methods were systematically evaluated using standard analytes spiked into both buffer and human plasma. We compared recovery, coverage, repeatability, matrix effects, selectivity and orthogonality of all methods tested for non-lipid metabolome in combination with reversed-phased and mixed-mode liquid chromatography mass spectrometry analysis (LC-MS). Our results confirmed wide selectivity and excellent precision of solvent precipitations, but revealed their high susceptibility to matrix effects. The use of all seven methods showed high overlap and redundancy which resulted in metabolite coverage increases of 34–80% depending on LC-MS method employed as compared to the best single extraction protocol (methanol/ethanol precipitation) despite 7x increase in MS analysis time and sample consumption. The most orthogonal methods to methanol-based precipitation were ion-exchange solid-phase extraction and liquid-liquid extraction using methyl-tertbutyl ether. Our results help facilitate rational design and selection of sample preparation methods and internal standards for global metabolomics.

The objective of global metabolomics is to analyze all small-molecular-weight species (≤1,500 Da) in a biological sample^1^. LC-MS is currently the method of choice for global metabolomics studies because it provides the highest metabolite coverage using a single analytical technique^2^. Typically, several hundred to thousand(s) metabolites can be detected in a single analysis^3^. The size of human metabolome is currently unknown, but is projected to exceed the conservative estimate of 4229 endogenous metabolites in concentrations spanning 11 orders of magnitude^4^. The most recent estimates predict 8500 endogenous metabolites^5^, and up to 40000 additional exogenous metabolites, such as drugs, additives and toxins that may be present in human samples^6^. Considering the typical coverage of untargeted metabolomics analysis, it is clear that metabolome complexity is overwhelming the capacity of modern metabolomics methods. Therefore, new strategies to increase metabolome coverage are required.

The most widely used protocol for global metabolomics of plasma is solvent precipitation using cold methanol or methanol/ethanol (1/1, v/v) with a plasma-to-solvent ratio of 1 to 3 or 4^7^,^8^,^9^,^10^. Cold solvent is added to minimize the extent of enzymatic conversion of metabolites and to precipitate the proteins. The removal of proteins from plasma also prevents protein build-up on LC column which improves LC column lifetime and significantly increases the number of detected metabolites through disruption of protein binding and minimizing number of signals originating from proteins^11^. Methanol and methanol/ethanol are the solvents of choice due to high metabolite coverage as shown by several studies^8^,^9^,^11^. However, the wide selectivity of such solvent-based precipitations results in highly complex samples which precludes the detection of lower abundance metabolites. Liquid-liquid extraction (LLE) using methyl-tertbutyl ether (MTBE) has become a popular alternative in recent years for its ability to provide good coverage of both polar and lipid metabolomes and compatibility with robotic systems^13^,^14^. In contrast, solid-phase extraction (SPE) methods are often avoided in global metabolomics of plasma due to their increased selectivity comparative to methanol-based extraction methods. SPE methods, thus, tend to decrease overall metabolite coverage^11^ but may improve data quality through improved repeatability^11^,^15^ and reduced matrix effects^12^,^16^. For instance, optimized HybridSPE™ successfully removed phospholipids in order to lower matrix effects while maintaining acceptable recoveries and repeatability^11^. In order to increase metabolome coverage beyond what can be achieved with methanol-based precipitations, multiple orthogonal extraction methods can be combined in a sequential manner as successfully shown in lipidomics where 2-fold improvement in coverage was achieved using different SPE fractionation approaches^7^,^17^. However, no similar sequential extraction approaches exist to date for non-lipid metabolome. To systematically design such sequential extraction protocol(s), it is necessary to directly compare coverage of various solvent precipitation, LLE and SPE methods. However, only a limited number of studies compared solvent precipitation methods to SPE and LLE to date^7^,^11^,^12^. Based on these published evaluations of extraction methods in real samples, a few limitations should be highlighted. None of these studies examine the orthogonality of SPE and MTBE methods to methanol-based methods in side-by-side fashion and comparison across the studies is not possible due to the different instrumentation and data processing strategies used. Most of these studies focus on metabolome coverage and extraction repeatability only, and no simultaneous evaluation of matrix effects and recovery in biological matrix has been performed to date. Recovery studies are crucial in order to design sequential extraction methods that are fully orthogonal and minimize spliting of the signal between multiple fractions. In addition, semi-quantitative comparisons of metabolite signal intensities between extraction methods can be misleading because variations in analyte signals due to matrix effects are not properly taken into account using the addition of stable isotope labeled (SIL) analytes^18^,^19^, fully isotopically-labeled complex matrices, or standard addition calibration. The latter approach was successfully employed to monitor and compare absolute recovery of sequential extraction by hybrid and mixed-mode SPE in untargeted metabolomics^20^. The underappreciated advantages of standard addition method become obvious when comparing different extraction methods. It is well-established that the slopes of calibration curves for biofluids originating from multiple populations can show significant differences^21^. Similarly, different matrix effects are expected in samples originating from extraction methods with different selectivity. In such cases, signal intensity changes may be driven by matrices alone leading to erroneous conclusions regarding the extraction performance. Although matrix effects are extensively studied in targeted bioanalysis, this issue has not been addressed in global metabolomics of biofluids except in one study where post-column infusion experiment was performed to identify region of significant ion suppression^16^. However, anecdotal evidence across multiple comparison studies shows potentially significant matrix effects with huge differences in signal intensity observed when using different extraction methods^15^,^20^,^22^. Therefore, the quantification of absolute recovery and matrix effects using a systematic set of standard analytes when evaluating multiple extraction methods is missing from comparisons to date, leaving a critical gap in our knowledge.

Following an extraction, the most frequently used LC separation in global metabolomics is the parallel use of C18 reversed-phase (RP) chromatography and hydrophilic interaction chromatography (HILIC) to achieve good coverage of non-polar and polar metabolome respectively^23^,^24^. More recently, mixed-mode chromatographic materials combining RP and ion-exchange mechanisms in low-bleed MS-compatible stationary phases provide improved retention of a broad spectrum of metabolites^25^,^26^. The major objective of this study was to perform the first side-by-side comparison of three conventional solvent precipitation methods to test the effect of small changes in solvent polarity (methanol, methanol-ethanol, and methanol-MTBE), one LLE (MTBE) method and three post-deproteinization SPE methods (C18, mixed cation-anion exchange (IEX) and divinylbenzene-pyrrolidone (PEP2)) for LC-MS metabolomics of human plasma. Absolute recovery and matrix effects for standard analytes were evaluated for all seven extraction methods using standard addition method. The repeatability and selectivity/orthogonality of extraction methods were compared using both targeted metabolites and on global basis in combination with RP and mixed-mode IEX/RP (Scherzo) LC-MS. These data pave the way for the rational selection of the best and most complementary extraction methods of the human plasma metabolome and clearly show the effect of using multiple sample preparation methods in a given study design on metabolome coverage.

## Methods

### Solvents and reagents

LC-MS grade solvents/mobile phase additives and analyte standards were purchased from Sigma-Aldrich (Oakville, ON, Canada) unless stated otherwise. ACTH (1–39) was obtained from Anaspec (Fremont, CA, USA). Norepinephrine (d_6_), cholic acid (d_4_), epinephrine (d_3_), dopamine (d_4_), melatonin (d_4_), 4-aminobutanoic acid (d_6_) and phenylalanine (d_5_) were obtained from CDN Isotopes (Point-Claire, QC, Canada), while ^13^C_6_- thyroxine was purchased from Toronto Research Chemicals (Toronto, ON, Canada). MTBE was bought from Fisher Scientific (Toronto, ON, Canada), while all phospholipids were obtained from Avanti Polar Lipids (Alabaster, Alabama, USA). Kynurenine and D-erythro-sphingosine (further mentioned as sphingosine for brevity) were purchased from Cayman Chemicals (Ann Arbor, MI, USA). Solid stationary phases (PEP2, ODS-C18 and divinylbenzene conjugated with sulfonic acid and quaternary amine moieties (IEX)) were obtained from Agela Technologies (Wilmington, DE, USA). Citrated pooled human plasma was obtained from Bioreclamation (Baltimore, MD, USA) and was collected in accordance with the company’s code of ethics. All reagents were of analytical or higher grade.

### Standard analyte mix

The chemical diversity of metabolome is enormous both in terms of polarity and charge^27^,^28^. Using predicted octanol/water partition values, metabolites in human plasma cover a polarity range from -5 (polyamines, amino acids) to 10 (fatty acids) to 35 (triacylglycerides)^27^. For the charge state, metabolites can be separated into acidic, basic, neutral and zwitterion classes. For instance, the study analyzing charge properties of 2553 non-lipid human metabolites from Human Metabolome Database, found that approximately 22% of metabolites are neutral, while 46.5 and 18.2% contain acidic carboxylic and phosphate groups, respectively. Basic aliphatic amines and aromatic heterocyclic nitrogen groups were found in 16 and 24.5% of non-lipid metabolites, while 13.8% of compounds were zwitterions^29^. The focus of current work is non-lipid metabolome, so standard metabolite selection was confined to metabolites with high to intermediate polarity typically found in blood plasma. A few lipids were also included in the mix to help in the assessment of matrix effects and method selectivity towards lipids, but systematic evaluation of extraction performance for lipids was beyond the scope of this study. Therefore, we evaluated extraction methods using standards with limited but systematic set of chemical properties that (i) resembled class composition of a target samples and included acids, bases, neutrals, zwitterions, lipids and small peptides, (ii) were systematic and scalable in terms of chemical properties (Log P range of −3.9 to 11.5, MW range of 105 to 900 Da), and (iii) amenable to the RP and mixed-mode LC-MS analytical methods employed in the study. All individual stock solutions were prepared in appropriate solvents as summarized in Supplementary Table 1, divided into aliquots and stored at below −70 °C, while working standards were prepared at appropriate concentrations prior to analysis. Standard mix was prepared at 5 μg/mL from appropriate stock solutions using 20% methanol unless otherwise specified.

### Extraction of analyte standard from buffer

The standard mix was prepared at 5 μg/mL of each compound in 25 mM ammonium acetate, pH 6.5 buffer. This high concentration was required to avoid non-specific adsorptive losses. Buffer composition was selected to obtain suitable pH and ionic strength for IEX stationary phases in order to achieve maximum recovery of analytes, while ensuring MS compatibility. The standard mix was extracted in six replicates by solvent-precipitation (methanol/ethanol (1/1, v/v), methanol, methanol/MTBE (1/1, v/v), liquid-liquid extraction (MTBE) and solid-phase extraction (PEP2, C18, IEX). In solvent precipitations and LLE, 100 μL of standard mix was extracted with 400 μL of ice-cold solvent, vortexed for 30 min and centrifuged for 15 min at 15000 × g. All steps were executed at 4 °C. After centrifugation, 350 μL of the upper layer was dried and stored at below −70 °C until analysis. For SPE, 100 μL of standard mix was loaded on a 3 mL SPE cartridge containing 100 mg (C18, IEX) or 60 mg (PEP2) sorbent. The cartridges were washed with 1 mL of sample buffer and eluted into glass tubes with 1.5 mL of elution solvent specific for every sorbent: C18 with 0.1% formic acid in 100% acetonitrile, PEP2 with 150 mM ammonium acetate, pH 6.8 in 94% methanol and IEX with 400 mM ammonium acetate pH 6.8 in 42% methanol. Eluted samples were evaporated to dryness under vacuum, and stored at below −70 °C. Before analysis, all samples were reconstituted in 10 μL of 20% methanol containing 2.5 mM ammonium acetate (pH 6.5), sonicated at ambient temperature for 5 min, vortexed for 10 min, diluted with 90 μL of 2.5 mM ammonium acetate, pH 6.5, sonicated and vortexed for 5 and 10 min and centrifuged at 15000 × g for 30 s.

### Extraction of plasma samples spiked with standard analytes

Solvent precipitations and LLE were carried out as described above using (i) the sample buffer (composed of 2% acetonitrile in 2.5 mM ammonium acetate pH 6.5) to obtain a blank extract for each method, (ii) plasma samples spiked with standard analytes to yield approximately 800 ng/ml before extraction and 100 ng/mL at LC-MS step in six replicates and (iii) unspiked plasma samples in 12 replicates to be pooled on per-method basis and used to build calibration curves for each method. Prior to SPE extraction, replicates of plasma and sample buffer were precipitated using methanol as described above, evaporated to dryness, reconstituted in the sample buffer, pooled as appropriate, and divided into replicates equivalent to 100 μL of plasma. Six of these replicates were spiked with the standard analytes at 800 ng/mL per each of three SPE methods. All samples were extracted by three SPE sorbents in parallel following the protocols described above to generate the sample sets similar to the one prepared for precipitation and LLE, i.e. blank extracts (i), spiked plasma extracts (ii), and unspiked plasma extracts (iii). All samples were dried under vacuum and stored at below −70 °C.

### Preparation of plasma extracts for LC-MS analysis

All plasma extracts were reconstituted in 30 μL of 20% methanol as described for standard analytes and further dissolved in 270 μL of 2.5 mM ammonium acetate. Standard addition calibration curves were prepared for each extraction method by adding 30 μL of the sample buffer or the mix of standard analytes to yield matrix calibration curve with 0 or 62.5, 125, 250 and 500 ng/mL, respectively. For the assessment of matrix effect, an external standard calibration curve in 2.5 mM ammonium acetate pH 6.5, 2% acetonitrile was also prepared in the same concentration range.

### LC-MS analysis

All extracts (10 μL injection volume) were analyzed on 1290 UPLC chromatograph (Agilent Technologies, Santa Clara, CA) using 3.0 μm, mixed-mode Scherzo SM-C18, 2 × 150 mm column (Imtakt, Portland, OR) and 1.8 μm Zorbax Eclipse octadecyl 2 × 200 mm column coupled to Agilent 6550 iFunnel Q-TOF mass spectrometer in positive and negative ESI in the mass range 100–1000 m/z. Additional details including LC-MS settings are provided in SI.

### Data analysis

TOF Quant software (version B.07.00 SP1, Agilent) was used for the determination of absolute recovery of standard metabolites from buffer and plasma. Raw data was extracted at 15 ppm mass accuracy, aligned within ±0.15 min retention time, integrated and corresponding adducts were summarized. Protonated and deprotonated ions were used for all other analytes in positive and negative ESI, respectively except for melatonin in positive ESI where sodiated adduct was also found. Quantitation was executed using external calibration curves in buffer and standard addition calibration in plasma. The recoveries of each analyte for each extraction method were hierarchically clustered using the Euclidian distance method with CIMminer online analysis at http://discover.nci.nih.gov/cimminer/oneMatrix.do^30^. The recoveries of analytes below 5 and above 80% were assigned to 0 and 100%, respectively for correct visualization. Matrix effect was calculated by dividing the peak area of an analyte in matrix calibration standard spiked post-extraction by the area in the calibration standard prepared in the sample buffer at the same analyte concentration and converting to percentage. The subtraction of endogenous signals was performed using signal obtained in unspiked plasma extracts. The final results reported for matrix effect represent the mean value obtained across four different concentrations tested for each analyte. Pooled QC samples for all target analytes in all analytical batches showed RSD ≤ 25%. QC data showed no evidence of analyte degradation in extracted plasma samples except possibly for histamine and sphingosine both of which showed systematic 20–30% decrease of signal intensity throughout the long analytical batches. For the global evaluation of the extraction methods, peak picking, deconvolution, alignment and integration were executed on Profinder (Agilent) with the following parameters: ion mass threshold of ±15 ppm, relative height of MS + 1/MS isotope abundance 15%, RT threshold ±0.15 min, minimum peak height 200 and 2000 counts for M + 1 and M peaks, respectively. The selectivity and repeatability analyses were carried out on Mass Profiler Professional (MPP, Agilent) with integration and binning parameters similar to Profinder, after removal of low quality metabolite signals that (i) were not at least 5x higher than the signal in blank and (ii) that were not found in at least 5 out of 6 replicates of a given extraction method. The data were manually verified and found to include 2–3% duplicate entries (a feature split between multiple entries by the peak picking algorithm). Therefore, the accuracy of putative metabolite coverage is ±3%. The orthogonality of extraction methods in global metabolomics approach was evaluated in a pairwise manner using the above high-quality data. Number of matched features for all possible paired combinations were used for hierarchical clustering using CIMiner online tool.

## Results and Discussion

Seven different extraction methods are compared based on the absolute recovery of standard analytes from buffer and human plasma, repeatability, selectivity and matrix effects in parallel with global LC-MS based metabolomics analysis. The overall experimental design is shown in Supplementary Figure 1. To the best of our knowledge, this is the first side-by-side comparison of the quality of sample preparation from blood plasma by conventional solvent precipitations (methanol-ethanol, methanol, methanol-MTBE), LLE (MTBE) and SPE (C18, IEX, PEP2) methods in a single study.

## Targeted Analysis

### Recovery, repeatability and selectivity of metabolite extraction from buffer

**Figure 1:**
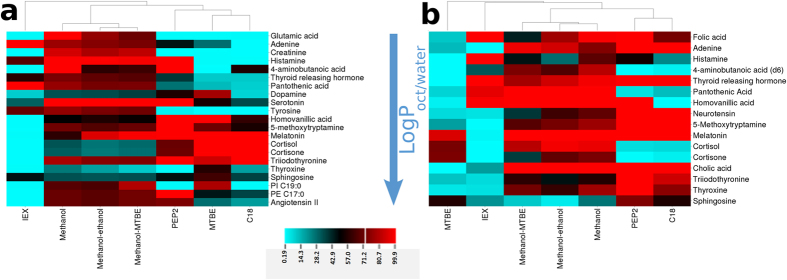
Hierarchical analyses and heat maps show the recovery of standard analytes from buffer (**a**) and human plasma (**b**). All extraction methods were hierarchically clustered using Euclidian distance method. The intensity of each cell represents range of recovery of an analyte relative to the initial amount spiked prior extraction. Recoveries below 5 and above 80% were assigned to 0 and 100%, for visualization purposes. The order of analytes corresponds to the increase in octanol-water partition coefficients, except for angiotensin II which did not have predicted value.

Analyte recovery is summarized in Fig. 1a and Supplementary Table 2. The analytes are listed by increasing logP values retrieved from Chemspider database predicted using ACD Laboratories algorithm. The extraction methods are arranged according to the results of hierarchical analysis. As expected, methanol, methanol/ethanol and methanol/MTBE extractions clustered closely together and provided the broadest coverage and the highest recovery across the wide range of metabolite classes tested. IEX provided high recovery only for polar charged metabolites, while MTBE provided high recovery for hydrophobic neutral metabolites. Among SPE methods, PEP2 provided broader metabolite coverage than C18 (Table 1), due to its ability to retain some of the polar metabolites. The highest selectivity in buffer was demonstrated by IEX, followed by C18 and MTBE. Moreover, IEX and C18/MTBE methods demonstrated little overlap, which can be exploited in sequential sample preparations for global and targeted metabolomics. None of the tested analytes exhibited ≥50% recovery in all of the extraction methods (Supplementary Table 2). Supplementary Table 4 summarizes the main performance characteristics of all extractions methods tested. The recovery of ≥80% is considered exhaustive and quantitative bioanalytical methods permit method precision of up to ≤20% RSD at lower limit of quantitation. However, very few metabolites can meet these most stringent criteria for any of the tested methods as shown in Supplementary Table 4. Global metabolomics methods are considered semi-quantitative, so applying more relaxed criteria of ≥50% recovery and ≤30% RSD is a reasonable compromise between method coverage and method performance. Using this criteria, methanol-based precipitations can provide acceptable performance for 17 out of 22 metabolites. Overall, metabolite recovery correlated to the predicted LogP values and the expected selectivity of the extraction methods. Neutral metabolites such as melatonin demonstrated the best quantitative (≥80%) recoveries amongst all standard metabolites. The best repeatability was demonstrated by methanol-based precipitations (Supplementary Table 4). SPE and LLE methods demonstrated lower repeatability then methanol blends with the poorest performance by MTBE and C18 SPE (Supplementary Table 4). This poor repeatability of MTBE is attributed to irreproducible partitioning of some metabolites between organic and aqueous phases, and was most pronounced for pantothenic acid, thyroxine and phenylalanine.

### Recovery, repeatability and selectivity of metabolite extraction from plasma

The high recovery (≥80%) was demonstrated by thyrotropin releasing hormone and melatonin in 6 out of 7 methods tested (Fig. 1b, Supplementary Table 3). Ionic compounds such as histamine, tyrosine and kynurenine with low molecular weight and LogP values demonstrated quantitative recovery in only 1 out of 7 methods. In addition, better recovery of triiodothyronine, thyroxine and the large peptide neurotensin (in contrast to tripeptide thyrotropin releasing hormone) was observed on RP SPE comparative to solvent based extractions (Supplementary Table 3A). The recovery of some analytes from plasma changed drastically in plasma *versus* buffer. The recovery of neutral compounds (cortisol, cortisone) by MTBE, PEP2 and C18 was decreased in plasma but the recovery in methanol-based solvents increased (Supplementary Tables 2A and 3A). This clearly shows the importance of performing recovery studies in biofluid matrix. The extraction repeatability (Supplementary Table 3B) showed similar trends to what was seen in buffer with methanol-based methods outperforming both SPE and LLE methods. However, SPE and LLE methods showed significant deterioration of method precision. For instance, MTBE method provided acceptable precision (≤30% RSD) for only melatonin, cortisol and triiodothyronine. Hierarchical clustering results shown in Fig. 1b confirm wide metabolite coverage of gold standard methanol-based solvent precipitation methods with high recovery across metabolite classes. The results also show the selectivity of MTBE is narrowed to uncharged species with LogP ≥ 1.4 and confirm orthogonal selectivity of IEX and MTBE methods previously observed for buffer. This can be used for the removal of hydrophobic compounds in sequential sample preparations. The effect of adding a second MTBE extraction step was also investigated to examine if this will further improve the metabolite recovery. The results of this experiment are shown in Supplementary Table 5. Only the recovery of folic acid and adenine increased when the second extraction step is included. For the remaining metabolites, no significant changes within experimental error where observed as expected theoretically due to high polarity of many of the metabolites within standard mix. Finally, methanol-based methods provide the most comprehensive and reproducible extraction of standard analytes from plasma (Fig. 1) as indicated by much lower mean RSD values than observed for other extractions (Supplementary Table 4). The more selective methods of SPE and LLE show good performance only for a narrow range of metabolites that are best suited to each extraction method depending on their polarity and charge characteristics. In both Supplementary Tables 2 and 3, some metabolites show recoveries above 120% in some of SPE methods. This result was surprising, considering the similar matrix composition of standard addition calibration and unknown samples, so it was investigated further. The first possibility considered was different adduct formation in buffer versus plasma. No such differences were found, so this was eliminated as contributing factor. Melatonin and melatonin d_4_ both had similar high recoveries in PEP2 and C18 SPE, which pointed to the fact this result may be due to co-suppression of spiked metabolites. The only compositional difference between calibration standards and samples used to evaluate recovery is the number of spiked metabolites present. Calibration standards were spiked after extraction and will therefore contain all metabolites of the standard mix, whereas the recovery samples were spiked before extraction and will remove or incompletely extract some of the metabolites from standard mix depending on the extraction method selectivity. This was further verified by re-analyzing the same extracts on longer chromatographic method (60 min analysis time), and proper quantitative recovery (80–120%) was obtained in all instances. These results clearly show that global metabolomics methods are extremely susceptible to matrix effects and that semi-quantitative performance of these methods can be affected by minor differences in matrix composition.

### Extraction preferences of standard analytes

Two groups of analytes emerged based on our recovery studies in buffer and plasma (Fig. 1). Analytes with LogP below 0.4 (above tyrosine) show poor recovery in MTBE and good recovery in methods suitable for extraction of polar species such as PEP2 and methanol-based solvent precipitations (Group I). The second group consists of less polar analytes (LogP ≥ 0.4) which demonstrate good recovery in MTBE, PEP2 and C18 (Group II). Interestingly, Group II analytes had recoveries ≥50% in most solvent and SPE methods in contrast to their recoveries from buffer. This difference in recovery is attributed to adsorptive losses in buffer. In the experiments with buffer we tried to minimize these losses by using high metabolite concentrations, but clearly adsorptive losses were still considerable especially for metabolites such as thyroxine, cortisone and cortisol. In conclusion, side-by-side systematic comparison of the absolute recovery of extraction methods was possible using standard addition calibration and showed clearly the critical importance of recovery determination in biofluids.

### Matrix effects

Matrix effects were evaluated using the post-extraction spike method at up to four different concentration levels. This evaluation was performed using both RP and mixed-mode LC-MS methods in positive and negative ESI. Ion suppression was observed for metabolites with lower LogP values (melatonin, 4-aminobutanoic acid, adenine and homovanillic acid) in methanol-based extractions in RP. Neutral analytes of intermediate polarity with LogP of 1.2 (cortisol and cortisone) were not affected in any extraction method, while signals from more hydrophobic metabolites (LogP ≥ 1.4, triiodothyronine and thyroxine) were enhanced in all solvent-based extracts, supressed in IEX and PEP2 and remained unaffected in C18 SPE. The suppression in solvent-based extractions in –ESI RP (Supplementary Figure 2, Supplementary Tables 6 and 7) affected wider range of analytes than in +ESI and extends toward neutral mid-hydrophobic analytes (cortisol and cortisone), while organic acids (folic, pantothenic, homovanillic and cholic) remained unaffected. The suppression of polar analytes in RP analysis in both positive and negative ESI is not surprising and could be explained by the co-elution of large number of un-retained metabolites. However, mixed-mode LC-MS analysis which is capable of chromatographically separating the majority of these charged species also shows very significant problems with ionization suppression and/or enhancement depending on the analyte and extraction method tested. Previous studies have also shown that HILIC methods are also highly susceptible to matrix effects even when using microextraction format^22^. Matrix effect could be partially decreased via improved resolution at LC-MS step by the decrease of stationary phase particle size (<2 μm) or drastic increase of column length and chromatography time to limits which may be impractical in real study^31^. Therefore, there is no simple solution to implement to address this major problem. Finally, methanol-based extracts demonstrated higher number of analytes affected by matrix effect. In contrast, the more selective MTBE and C18 SPE methods showed matrix effects for fewer metabolites and less pronounced extent of suppression/enhancement if matrix effects were present (Table 1, Supplementary Figure 2). The higher suppression matrix effect observed for methanol blends is most likely caused by higher matrix complexity as compared to MTBE and C18 SPE methods which are more selective as shown by our recovery experiments. Overall, the results of this comparison show that matrix effects pose a significant challenge in all extraction protocols and can have significant impact on biomarker discovery efforts. Additional ways to reduce and evaluate matrix effects during such studies should be explored and implemented. Table 1 also shows that the analyses performed using mixed-mode chromatography are more susceptible to signal enhancement. The observed enhancement of signal response (for example, triiodothyronine and thyroxine in Scherzo analysis (Supplementary Figure 2) may be explained by: (i) true matrix effect; (ii) the presence of co-eluting isobaric contaminants whose signals were mistakenly included due to insufficient resolution of QTOF; (iii) formation of significant amount of adducts in buffer but not in matrix calibration points and/or (iv) limited solubility of standard analytes in buffer calibration samples versus plasma extracts. To evaluate possibility (ii), the matrix effect experiment was repeated for +ESI Scherzo LC analysis using Orbitrap Velos^TM^ mass spectrometer with mass resolution of 100,000. The same enhancement results were observed, so we can conclude that the cause of observed ion enhancement is not co-elution of species with similar m/z to the analytes of interest. Next, Na^+^ and NH4^+^ adduct formation was compared for buffer samples *versus* plasma extract samples. No significant differences in adduct formation were found for any analytes except for melatonin where sodiated adduct with the intensity similar to protonated ions was formed in buffer calibration points. To correct for sodiated adduct formation, total area of sodiated and protonated ions was used in calculations of matrix effect for melatonin. Finally, the observation of large differences between matrix effects in Scherzo and RP despite identical sample preparation protocols between these two methods exclude the involvement of partial solubility. Thus, it is plausible to conclude that the enhancement effect observed in mixed-mode LC analysis (and occasionally in RP analysis) is based on true difference in ionization process between plasma-based and buffer-based samples. This is further supported by the fact that for both analytes that exhibit ion enhancement, their isotopically labelled standards also confirm the same extent of enhancement showing high quality of the collected data.

### Selection of internal standards for global metabolomics

Our recovery and matrix effects results can be used to guide the selection of the best internal standards for quality control for human plasma metabolomics. Standards that show no susceptibility to matrix effects make ideal internal standards (as SIL analogues) to spike before extraction in order to monitor extraction recovery. These include 5-methoxytryptamine, folic acid, thyrothropin releasing hormone and cortisol for +ESI RP; and pantothenic and cholic acids for −ESI RP all of which showed no matrix effects across all seven extraction methods tested as shown in Supplementary Table 6. These can be supplemented with additional standards spiked post extraction to monitor for matrix effects such as SIL analogues of adenine and thyroxine for ±ESI RP; neurotensin, melatonin, thyroxine and cortisol for +ESI Scherzo and homovanillic acid, melatonin and thyroxine -ESI Scherzo. These analytes show large differences in matrix effects between different extraction methods as shown in Supplementary Figure 2 which suggests their ionization is susceptible to presence of possible co-eluting interferences, and do not overlap with proposed recovery standards. The use of matrix effect standards is suggested to evaluate relative matrix effects between individual samples but it should be noted that they would only reflect the extent of ion suppression at that specific moment of chromatographic run. Finally, the above internal standard suggestions are valid for the exact extraction methods, plasma loading and LC methods tested in this study. Further testing is required to extrapolate the use of these specific standards to other experimental conditions. In general, for any combination of extraction method and LC-MS analysis, standards with high recovery in that extraction method and no matrix effects across all extractions for the chosen LC-MS method would make ideal recovery standards, while standards highly susceptible to ion suppression/enhancement would make useful internal standards for monitoring of matrix effects across individual samples.

## Global Metabolite Analysis

**Figure 2:**
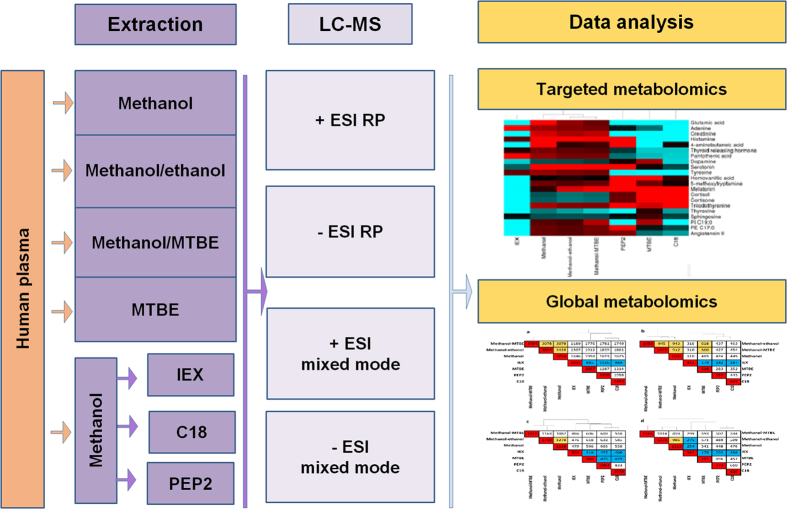
Overview of experimental design to compare seven extraction methods for untargeted metabolomics analysis of human plasma.

Seven extraction protocols were compared using four LC-MS analyses in order to assess metabolite coverage, extraction repeatability and method overlap (orthogonality) as shown in Fig. 2. Table 2 summarizes metabolite coverage and extraction repeatability of all extraction methods tested. As expected, the highest number of putative metabolites was extracted by methanol-based solvent precipitation methods, with the highest number of putative metabolites (3804) detected for methanol/ethanol. The analysis of organic MTBE fraction resulted in 2887 putative metabolites as revealed by +ESI RP analysis. Approximately 30% less metabolite features were detected in C18 and PEP2 SPE extracts while only 1835 putative metabolites were observed for IEX SPE. The table also shows median RSD of signals across all extraction methods for each LC-MS analysis. Methanol-ethanol and methanol extractions demonstrated the best repeatability versus all other extraction methods independently of LC-MS method employed. PEP2 and IEX had acceptable repeatability for global metabolomics. On the other hand, MTBE and C18 extraction methods demonstrated the worst repeatability independent of LC-MS analysis (Table 2). The high proportion of irreproducible features in these two methods requires the application of rigorous quality controls and in-depth investigation for the sources of such irreproducibility. Previous C18 SPE studies for plasma metabolomics indicate conflicting evidence regarding C18 repeatability for this application. In the first study on this topic, Michopoulos *et al*. showed 48% and 55% of features detected in C18 SPE and methanol precipitation had RSD ≤30% respectively, which implied both methods have similar repeatability^15^. Rico *et al*. also observed similar repeatability between methanol and C18 SPE with approximately 80% of features which met 30% RSD criteria for both methods^11^. Our results show that only 42% of features extracted by C18 SPE met the repeatability criteria which is consistent with Michopoulos *et al*. study^15^. However, our results also show vast superiority of methanol repeatability where 92% of features were highly repeatable with RSD ≤ 30% for n = 6 extraction replicates. Considering this discrepancy across the studies for C18 SPE, further investigation of the factors affecting repeatability is required. Such contributing factors may include lack of automation used in our study and the exact selection of sorbent characteristics and wash/elution conditions. For instance, our study employed acetonitrile, whereas both Michopoulos *et al*. and Rico *et al*. used methanol as elution solvent which may have contributed to the poor precision^11^,^15^. Our MTBE results are in contrast to good precision obtained when using MTBE with in-vial dual extraction method where ≥ 80% of features had RSD ≤ 30% for n = 3 extraction replicates^14^. The same authors observed poor precision of MTBE LLE with evaporation/reconstitution step whereby only 56% of detected features exhibited RSD ≤ 30%. The latter result is consistent with the results of the current study where evaporation/reconstitution step was employed. During their evaluation of optimum method for lipidomics, Sarafian *et al*. also showed poor repeatability of MTBE in comparison to methanol with a similar 2-fold deterioration of mean RSD and the numbers of highly reproducible lipids^32^ consistent with what was observed in current study. During further investigation of MTBE extraction repeatability, the repeatability of solvent pipetting was investigated and found not to be significant contributing factor to overall method performance. Next, the +ESI RP LC-MS analysis of newly-prepared aqueous and organic layers obtained after MTBE extraction found that aqueous extracts had repeatability similar to that of methanol (median RSD of 15.7% for MTBE aqueous) versus 16.0% RSD for methanol obtained during this follow-up experiment. In contrast, organic extracts had median RSD of 36.4% which is consistent with 37.9% median RSD obtained in our initial experiment presented in Table 2. Detailed investigation of this data showed clear dependence of RSD on retention time: large proportion of peaks eluting with retention time of >20 min had RSDs greater than 50% in both methanol and MTBE extracts. Methanol had large number of peaks with retention time <20 min which exhibited good repeatability, which resulted in good median RSDs observed for the global metabolomics data. MTBE, on the other hand, had only small number of metabolites eluting with retention time <20 min, and very high proportion of metabolites with retention time >20 min, which resulted in overall higher median RSDs observed in Table 2. Based on this evidence, it is believed that poor MTBE repeatability observed in our study may not arise from the extraction method itself, but from poor match between the composition of MTBE extract and RP and mixed-mode LC separation methods employed in this study which would not adequately separate lipids extracted by MTBE. This conclusion is further supported by the observed increase in median RSD from 35.4 to 50.9% when one-step MTBE and two-step MTBE extractions were compared. Although 2-step extraction increased recovery of few mid-polar metabolites, this also increased the extent of co-elution thus causing further deterioration in RSD.

**Figure 3:**
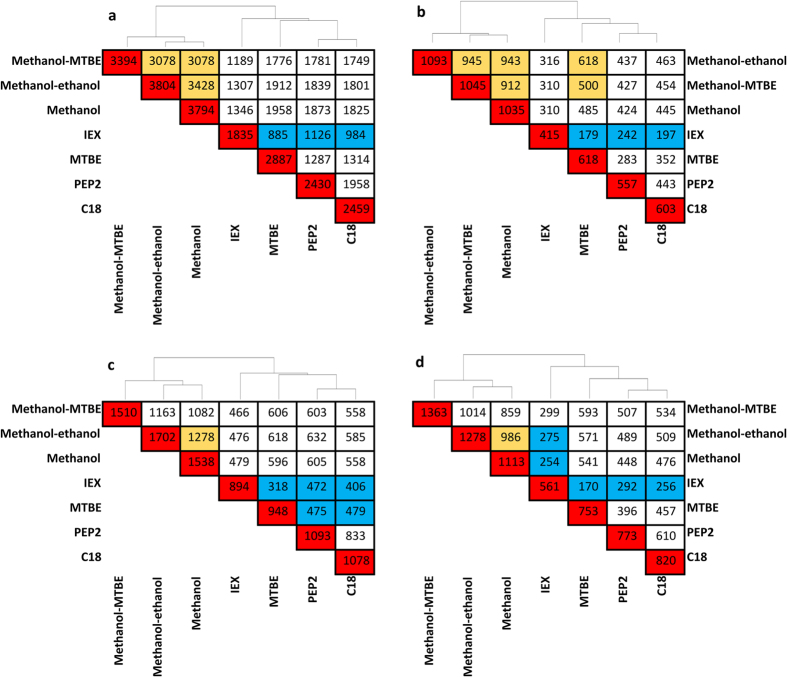
Hierarchical clustering of the number of putative metabolites detected in plasma extracts and pairwise overlap coverage of seven extraction methods. Samples were analyzed using +ESI RP (**a**) −ESI RP (**b**), +ESI Scherzo (**c**) and −ESI Scherzo (**d**). Red colour boxes located across the diagonal show the total number of putative metabolites detected with that extraction method from plasma. Yellow, white and cyan blue colors designate high (99.9–80% overlap), medium (79.9–50.0%) and low (50.0–0% overlap) of putative metabolite populations observed by the two extraction methods specified. Therefore, the methods indicated with cyan blue boxes are the most orthogonal pairs of methods across all of the seven extraction methods tested.

**Figure 4:**
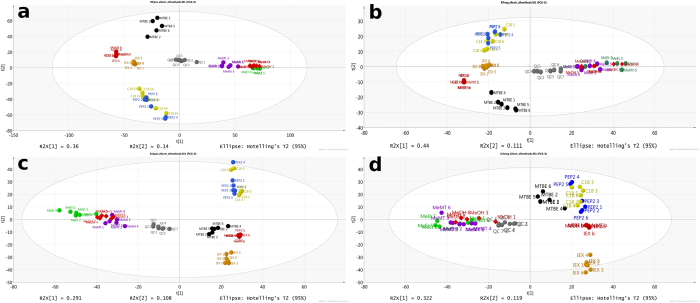
PCA analysis of seven extraction methods and quality control samples analyzed on four LC-MS methods (**a,b** – reversed phase analysis; **c,d** – Scherzo mixed-mode analysis in positive and negative ESI, respectively) executed on all metabolites that satisfy criteria described in main text (not present in blank, and present in minimum of 5 out of 6 replicates of at least one extraction method). The graph displays colored spheres for blank (red), QC (dark grey), IEX (dark gold), C18 (yellow), PEP2 (blue), MTBE (black), methanol-MTBE (violet), methanol-ethanol (green) extraction replicates and red diamonds for methanol extraction replicates. The plots show the top two principal components. Numbering of replicates corresponds to their sequential injection order in a given LC-MS analysis. Analysis was executed using multivariate analysis software SIMCA (v 14.1.64, Umetrics, San Jose, CA, USA) after Pareto scaling.

Further analysis of Table 2 across LC-MS methods demonstrated inferior reproducibility of Scherzo analysis comparative to RP. This is attributed to lower resolution of Scherzo column (larger particle size than RP column) and larger matrix effect than in RP as shown in Matrix Effects section. Finally, hierarchical analysis was performed to determine pairwise orthogonality of each of the methods tested. The results are shown in Fig. 3. The hierarchical clustering confirms the high orthogonality of IEX and MTBE to other methods observed in targeted analysis, similarity between methanol-based extractions and similarity between C18 and PEP2 SPE methods. Our orthogonality results for methanol and C18 SPE (1825/2459 putative metabolites =74% overlap using data shown in Fig. 3) are consistent with what was reported by Rico *et al*. who observed 58–68% overlap between the two methods and ability to detect 600 additional features when comparing SPE to methanol precipitation^10^. Using +ESI RP analysis, total of 5853 non-redundant putative metabolite features were detected across all seven extraction methods tested. This represents only 54% improvement over the single best extraction method of methanol/ethanol (3804 putative metabolites) or methanol (3795 putative metabolites). Therefore, 7x increase in MS analysis time and the use of LLE and SPE with widely different selectivity mechanisms did not provide a huge boost in our ability to detect low abundance metabolome. Similar results were observed for other LC-MS methods where the increases were 34% (−ESI RP LC-MS), 80% (+ESI mixed mode LC-MS) and 74% (−ESI mixed mode LC-MS). These results clearly show that simply using multiple extraction methods in parallel is not the best way to increase metabolite coverage and that sequential extractions should be explored to further boost metabolome coverage. Figure 4 shows principal component analysis results for all extraction and LC-MS methods further illustrating that IEX and MTBE are the most complementary methods to methanol-based solvent precipitation.

## Conclusions

For the first time, absolute analyte recoveries and matrix effects in plasma were systematically assessed for seven solvent precipitations, LLE and SPE methods using standard addition calibration. In addition, method repeatability, orthogonality and metabolome coverage were compared in combination with two reversed-phased and mixed-mode LC-MS methods. Our results confirm wide selectivity of methanol-based precipitation methods versus LLE and SPE, with the best results observed using methanol or methanol/ethanol as shown in Table 3. However, methanol-based methods suffer from severe matrix effects which negatively impacts data quality and may result in inaccurate selection of tentative biomarkers. We also show that IEX and PEP2 SPE provide acceptable performance for global metabolomics studies of plasma, and can be employed depending on the desired coverage of the metabolome for a given application. Our analysis platform revealed high orthogonality of MTBE and IEX to each other and other methods, providing the possibility of increased metabolome coverage via sequential application of these methods.

## Additional Information

**How to cite this article**: Sitnikov, D. G. *et al*. Systematic Assessment of Seven Solvent and Solid-Phase Extraction Methods for Metabolomics Analysis of Human Plasma by LC-MS. *Sci. Rep.* **6**, 38885; doi: 10.1038/srep38885 (2016).

**Publisher's note:** Springer Nature remains neutral with regard to jurisdictional claims in published maps and institutional affiliations.

## Supplementary Material

Supplementary Information

## Figures and Tables

**Table 1:** Summary of total number of standard analytes which experienced matrix effect (suppression or enhancement) across different extraction methods in combination with either RP or mixed-mode LC-MS analysis.

Evaluation of matrix effects	RP LC-MS analysis							Scherzo mixed-mode LC-MS analysis						
	Methanol- ethanol	Methanol-MTBE	Methanol	MTBE	IEX	PEP2	C18	Methanol- ethanol	Methanol-MTBE	Methanol	MTBE	IEX	PEP2	C18
Suppressed (+ESI)	6	6	6	4	7	9	5	4	4	4	4	4	7	5
Enhanced (+ESI)	5	5	5	4	2	4	2	11	11	11	7	7	3	2
Total affected (+ESI)	11	11	11	8	9	13	7	15	15	15	11	11	10	7
Total unaffected (+ESI)	6	6	6	12	7	4	10	2	2	1	8	4	7	10
Suppressed (−ESI)	3	3	5	0	1	1	1	7	4	9	1	10	5	3
Enhanced (−ESI)	2	2	2	2	4	7	8	4	3	2	5	1	4	2
Total affected (−ESI)	5	5	7	2	5	8	9	11	7	11	6	11	9	5
Total unaffected (−ESI)	5	5	3	10	9	6	5	2	6	2	8	1	4	9

Analytes (n = 24, including SIL analogues for some of the metabolites) were counted if they were detected in buffer and at least one of the post-extraction spiked calibration standards. For metabolites detected at all concentration levels the mean matrix effect obtained across all concentration levels is reported. A metabolite was considered to be enhanced if its matrix effect ratio exceeded 120% or suppressed if it was less than 80%. The species that were not detected in either matrix were not counted, because matrix effect could not be properly determined for such cases. Supplementary Table 7 shows full results for matrix effect evaluation.

**Table 2:** Metabolite coverage and repeatability of extraction methods assessed by global metabolomics analysis.

Extraction method	+ESI RP			−ESI RP			+ESI Scherzo			−ESI Scherzo		
	M	Median % RSD	M_30_	M	Median % RSD	M_30_	M	Median % RSD	M_30_	M	Median % RSD	M_30_
Methanol-ethanol	3804	12.8	3306	1093	13.4	1051	1702	22.3	1087	1278	18.7	930
Methanol-MTBE	3394	17.7	2389	1055	12.7	849	1510	25.2	877	1363	21	897
Methanol	3795	11.5	3483	1035	11.4	940	1538	19	1089	1113	17.9	802
IEX	1835	17.5	1406	415	12.5	364	894	23.2	571	561	23.1	373
MTBE	2887	37.9	1037	618	26.9	362	948	39.1	326	753	31.7	345
PEP2	2430	21	1635	557	14.5	444	1093	24.9	651	773	21.5	498
C18	2459	34.6	1032	603	22.7	394	1078	41.4	318	820	33.2	357
Total metabolome coverage	5853			1466			3072			2229		

This table shows total number of putative metabolites (M) detected in minimum 5 out of 6 extraction replicates analyzed using RP or Scherzo mixed-mode LC-MS after removal of features present in blank extracts, median RSD of signal intensity across all putative metabolite features detected in the extraction method and the number of metabolites for which extraction method was highly repeatable with RSD ≤ 30% for n = 6 independent extractions (M30). RSD for each putative metabolite feature was calculated using raw signal intensities in extraction replicates (n = 6). Number of features with RSD ≤ 30% (between replicates) represent high quality features that could be used for biomarker discovery and pathway analysis in global metabolomics projects.

**Table 3:** Summary of method performance for extraction of plasma.

Extraction method	Recovery	Matrix effects	Repeat ability	Metabolome coverage
Methanol/ethanol	+++	++	+++	++++
Methanol	++++	++	++++	++++
Methanol/MTBE	+++	++	+++	+++
MTBE	++	++++	+	++
C18	+++	++++	+	++
PEP2	+++	++	++	++
IEX	+	+++	++	+

Number of pluses represent the scoring of method performance where + is the worst and ++++ is the best.
