# Supplementary material for: Systematic Assessment of Seven Solvent and Solid-Phase Extraction Methods for Metabolomics Analysis of Human Plasma by LC-MS
Source: Sci Rep. 2016 Dec 21;6:38885. doi: 10.1038/srep38885 (PMC5175266; doi:10.1038/srep38885)
Supplement: Supplementary Information [file srep38885-s1.pdf]

Systematic Assessment of Seven Solvent and Solid-Phase Extraction Methods for Metabolomics Analysis of Human Plasma by LC-MS

Dmitri G. Sitnikov, Cian S. Monnin and Dajana Vuckovic*

Department of Chemistry and Biochemistry, Concordia University, Loyola Campus, 7141 Sherbrooke St. W. Montréal, Québec, Canada, H4B 1R6

SUPPLEMENTARY MATERIALS

EXPERIMENTAL – SUPPLEMENTARY METHODS

Plasma extractions were analyzed on C18 or Scherzo columns coupled to QTOF via positive and negative ESI. Therefore, each sample was analyzed in four LC-MS modes: (i) reversed-phase C18 UPLC in positive and negative ESI and, (ii) mixed reversed-phase/ion-exchange Scherzo HPLC in positive and negative ESI. Buffer extractions were analyzed on Orbitrap Velos^{TM} mass spectrometer in positive ESI.

LC-MS reversed phase method for the analysis of extractions in plasma

C18 separation was executed at 0.4 mL/min flow rate at 35°C using binary solvent system consisting of 0.1% formic acid, 2% acetonitrile in water (solvent A) and 0.1 % formic acid in 100% acetonitrile (solvent B). For LC-MS analysis in negative ESI, concentration of formic acid in both solvents was reduced to 0.05 %. The following gradient was used for separation: 2 % B for 3 min, then increase of B from 2 to 100 % for 20 min followed by 2 min hold at 100 % B and 4 min of re-equilibration to 100 % A.

Positive ESI QTOF settings were: capillary voltage of 3800 V for the entire run and nozzle voltage of 200 V for the first 4 min and 1500 V from 4th to 28th min of the analysis. In negative ESI, capillary and nozzle voltages were set to 3500 and 500 V, respectively during the first 5.5 minutes of run and to 4200 and 800 V, respectively between 5.5 and 29th minutes. For both positive and negative ESI, drying and sheath gas temperatures were set to 250 and 275°C and flow rates to 15 and 12 L/minutes, respectively. Nebulizer pressure was set to 30 psig and fragmentor voltage to 175 V. Data was acquired in both centroid and profile mode at the rate of 3 spectra per second in the extended dynamic range mode (2 GHz). Resolution of 12,000 FWHM (full width at half maximum) at m/z 121 and 24,000 FWHM at m/z 922 was achieved. To assure the desired mass accuracy of recorded ions, continuous internal calibration was executed using signals at m/z 121.0509 (protonated purine) and m/z 922.0098 (protonated hexakis (1H, 1H, 3H-tetrafluoropropoxy) phosphazine (HP-921)) in positive ion mode. For negative ESI analysis, ions with m/z 119.0363 (deprotonated purine) and m/z 966.0007 (formate adduct of HP-921) were used.

LC-MS mixed-mode method for the analysis of extractions in plasma

The stationary phase of Scherzo SM-C18 HPLC column is composed of C18 alkyl and weak cation and anion moieties. Therefore, solvent was supplemented with ammonium acetate buffer to execute mixed mode (reversed-phase/ion exchange) chromatography and elute ionic species from the column. The binary solvent system consisted of 2 mM ammonium acetate buffer, pH 3.5 in 2% acetonitrile and 98% of water (solvent A) and 100 mM ammonium acetate buffer, pH 3.5 in 96% acetonitrile and 4% of water (solvent B). The samples were separated at 0.22 mL/min flow rate at 35°C using the following gradient: 0 % B for the first 2 min, increase of B from 0 to 20% between 2nd and 10th min, increase of B from 20 to 100% between 10th and 25th min, isocratic hold at 100% B for 6 min and 6 min of re-equilibration to 100% A. In positive ESI, the capillary voltage was kept at 3500 V for the entire run, while nozzle voltage was set to 200 V for the first 5 minutes of run and to 800 V between 5th and 35th minutes. In negative ESI, nozzle and capillary voltages were held at 250 V and 3750 V for the entire run. All other MS settings were the same as described for LC-MS RP in previous section.

For targeted analysis, all samples were analyzed in random order with QC samples (mixture of all analyzed samples (iii) loaded at each 11th injection. XICs were extracted with 15 ppm accuracy and aligned within ± 0.15 minutes retention time interval. Metabolite identity was confirmed by the match of retention time and m/z between signals in analyzed samples and signals in calibration points. The concentrations of standard analytes were quantitated using external calibration curves obtained as described in materials and methods. For both targeted and global analyses, at least 10 runs of QC samples preceded batch runs in order to stabilize chromatography and ESI performance. For the global analysis, to ensure confident comparisons across extraction methods in global metabolomics analysis, samples from solvent, LLE and SPE extractions were analyzed side by side in 6 randomly organized sub-batches containing a single replicate from each of 7 extractions and one QC

sample. In order to analyze seven blank samples (type i) from seven methods in six sub-batches, five of sub-batches comprised one and the sixth sub-batch – two blank samples. PCA was performed using SIMCA 14 (Umetrics, Sweden) on all high-quality data described in Methods section, after Pareto scaling. PCA was used to verify the stability of LC-MS signals throughout the analysis by checking the clustering of QC signals for each analytical batch (Supplementary Figure 3). It can also be used to visualize repeatability and similarity between different extraction methods.

LC-MS mixed-mode method for the analysis of extractions in buffer

The column and buffer composition were identical to the analysis in plasma. Samples were separated at 0.22 mL/min flow rate at 35 ^{0}C using the following gradient: 0 % B for the first 2 min, increase of B from 0 to 100% between 2nd and 18th min, isocratic hold at 100% B for 6 min and 7 min of re-equilibration to 100 % A. Column was coupled to Orbitrap Velos^{TM} mass spectrometer in positive ESI, at the following settings: ESI voltage 3.25 kV, heater and capillary temperature at 350 ^{0}C, sheet gas, auxillary gas and sweep gas flow rates at 40, 10 and 0 arbitrary units, respectively; detection of signals was executed in the range 50-1000 m/z at 250 ms activation time, normalized collision energy 35 V, resolution 60,000 and mass accuracy 10 ppm. Deconvolution of raw data and quantitative analysis was executed using Xcalibur ^{TM}, v. 2.0 (Thermo Fisher Scientific, Waltham, MA, USA) and statistical analysis in Microsoft Excel unless otherwise specified.

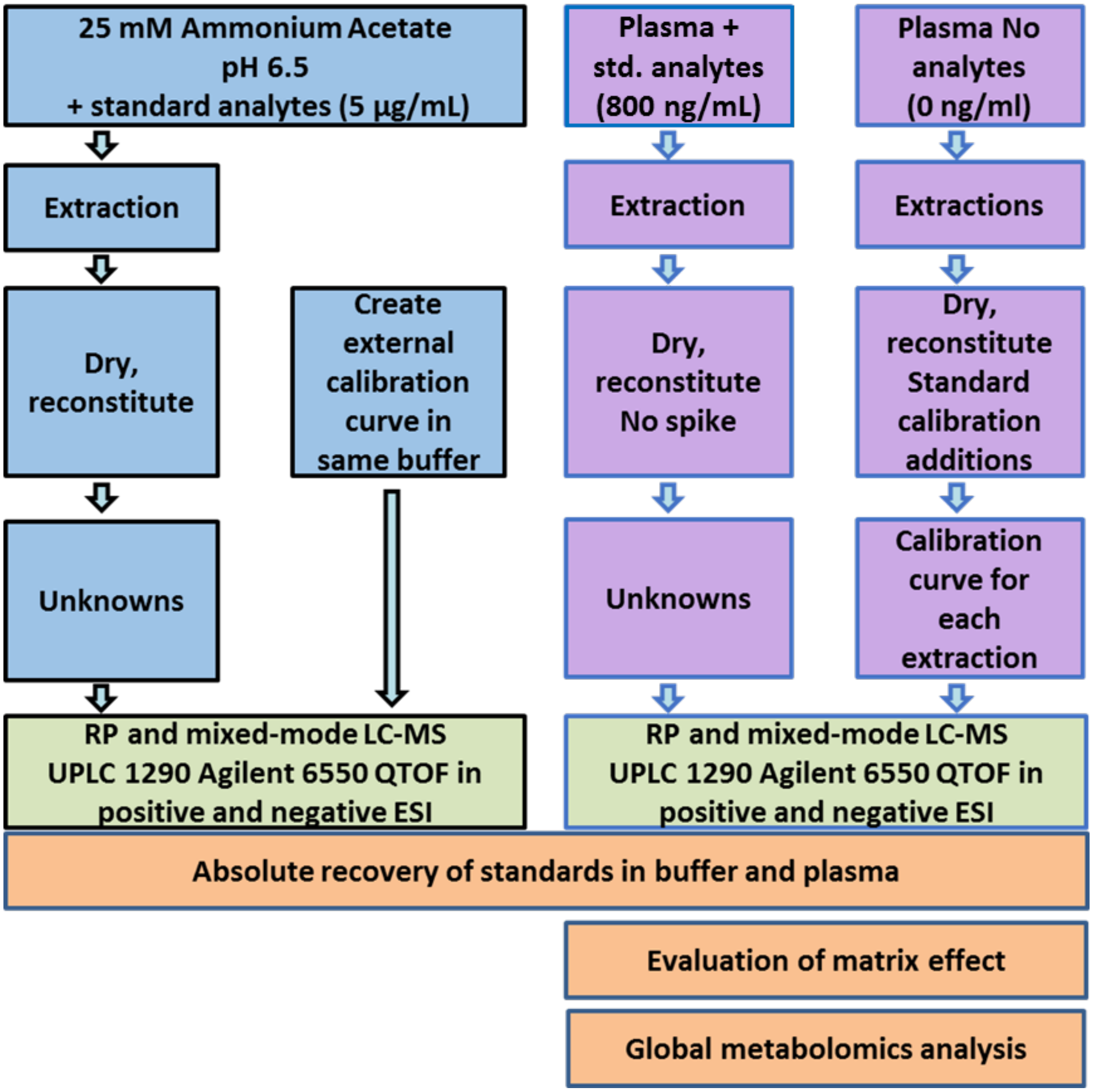

Supplementary Figure 1. Study design for the targeted and global metabolomics analysis of extraction methods. Blue and purple colors designate experiments in buffer and blood plasma, respectively. Green color designates LC-MS analyses step executed on RP (reversed phase) and mixed mode (weak anion/weak cation/RP) mode columns. Peach color designates major data analysis blocks.

Target analyte	Chemical group	Monoisotopic mass	RT on RP column (min)	RT on Scherzo column (min)	ACD/LogP	Buffer composition in the Individual stock
**4-aminobutanoic acid**	Zwitterion	103.0633	0.7	1.4	-0.9	50% methanol, 2 mM AmAc
**4-aminobutanoic acid (d6)**	Zwitterion	109.1043	0.6	1.4	-0.9	50% methanol, 2 mM AmAc
**5-methoxytryptamine**	Zwitterion	190.1106	6.6	8.6	1.3	Water
**ACTH**	Zwitterion	4508.0410	ND	ND	N/A	Water
**Adenine**	Positive	135.0545	0.9	2.8	-2.1	Water
**Cholic acid**	Negative	408.2875	13.2(-ve)	19.1 (-ve)	2.3	0.1% NH_4OH
**Cholic acid (d4)**	Negative	412.3167	13.2(-ve)	19.1 (-ve)	2.3	0.1% NH_4OH
**Cortisol**	Neutral	362.2093	10.7	16.8	1.4	50% methanol
**Cortisol (d4)**	Neutral	366.2366	10.7	16.8	1.4	50% methanol
**Cortisone**	Neutral	360.1937	10.8	16.9	1.4	50% methanol
**Creatinine**	Zwitterion	113.0589	0.7	1.6	-1.6	50% methanol
**Sphingosine**	Zwitterion	299.2824	14.6	20.4	6.4	50% methanol
**Dopamine**	Positive	153.0790	0.7	1.7	0.1	20% methanol, 0.1 % FA
**Dopamine (d4)**	Positive	157.1063	0.7	1.7	0.1	See Dopamine
**Epinephrine**	Negative	183.0895	ND	ND	-0.6	See Dopamine
**Epinephrine (d3)**	Negative	186.1100	ND	ND	-0.6	See Dopamine
**Folic acid**	Zwitterion	441.1397	6.6	14.7	-3	0.1% NH_4OH, 50% methanol
**Glutamic acid**	Zwitterion	147.0531	1.1	6.4	-3.9	water
**Histamine**	Zwitterion	111.0796	1.5	2	-0.7	water
**Homovanillic acid**	Negative	182.0579	7.3	14.2	1.1	as Folic acid
**Homovanillic acid (d3)**	Negative	185.0784	7.3	14.2	1.1	as Folic acid
**Kynurenine**	Zwitterion	208.0848	2.9	5.8	1.1	50% methanol, 0.1 % FA
**Melatonin**	Neutral	232.1210	9.2	15.5	1.2	50% methanol
**Melatonin (d4)**	Neutral	236.1485	9.2	15.5	1.2	50% methanol
**Neurotensin**	Zwitterion	1671.9097	8.2	12.7	N/A	0.1 % FA
**Norepinephrine**	Positive	169.0740	ND	ND	-0.9	see epinephrine
**Norepinephrine (d6)**	Positive	175.1149	ND	ND	-0.9	see epinephrine
**Pantothenic acid**	Negative	219.1107	5.0	8.6	-0.4	See Folic
**PC (19:0/19:0)**	Positive	818.5540	ND	ND	11.5	90% methanol, 10% IPA
**PE (17:0/17:0)**	Positive	719.5465	21.1	ND	11.5	
**Phenylalanine (d5)**	Zwitterion	170.1155	3.0	5.3	1.1	See Dopamine
**Angiotensin II**	Zwitterion	1045.5345	8.9	ND	N/A	See Dopamine
**PI (18:3/22:4)**	Neutral	908.5391	21.4	ND	11.5	See PC
**Serotonin**	Zwitterion	176.0950	2.5	6.8	0.2	See Dopamine
**Thyroxine**	Negative	776.6867	11.8	18.2	5.9	50-50 DMSO-methanol
**Thyroxine (13C6)**	Negative	782.6860	11.8	18.2	5.9	
**Triiodothyronine**	Negative	650.7900	11	17.2	5.1	
**Tyrosine**	Zwitterion	181.0739	1.4	3	0.4	See Dopamine
**Thyrotropin releasing hormone**	Positive	362.1703	1.5	2.6	N/A	See Dopamine

Supplementary Table 1 Standard analytes used in the study. Monoisotopic masses and predicted ACD/LogP values were obtained from ChemSpider (http://www.chemspider.com). LogP values predicted by ACDLabs algorithm (See Table 1 in the text for details). LogP predicted values could not be found for some metabolites in ChemSpider database and these entries are shown as not available (N/A) in the table. Retention times provided are for 500 ng/mL buffer calibration (= highest concentration analyzed) point analyzed in either ESI mode. ND stands for not detected and (-ve) designates analytes detected at negative ESI mode. ACTH, epinephrine, norepinephrine and PC (19:0/19:0) were not detected in any conditions and are removed from subsequent tables. For details on the usage and fate of analytes in experiments see Supplementary Tables 2, 3 and 7. Other abbreviations: AmAc - ammonium acetate, FA- formic acid, NH_4OH-ammonium hydroxide, IPA-isopropanol, DMSO-dimethylsulfoxide.

Analytes	Methanol-Ethanol	Methanol	Methanol-MTBE	MTBE	C18	PEP2	IEX
**A. Recovery (% of input) of standard analytes from buffer**							
**Glutamic acid**	79.0	106.3	70.8	ND	ND	25.7	73.4
**Tyrosine**	73.9	79.1	71.4	ND	ND	2.3	70.0
**Creatinine**	83.7	91.6	87.6	ND	ND	ND	ND
**Thyrotropin releasing hormone**	68.8	74.1	66.9	10.4	10.2	39.3	57.7
**Pantothenic Acid**	73.2	79.5	73.9	13.1	9.1	27.7	120.9
**Histamine**	112.2	122.4	98.7	ND	ND	133.6	67.0
**4-aminobutanoic acid**	58.6	123.7	61.7	ND	55.5	141.9	ND
**Adenine**	75.9	80.4	73.5	44.6	ND	46.1	108.4
**Dopamine**	35.2	34.4	38.0	82.0	8.1	55.5	9.8
**Serotonin**	106.2	117.5	110.5	54.7	35.3	107.0	31.2
**Homovanillic acid**	56.0	50.3	51.6	102.7	60.0	122.2	ND
**Phenylalanine (d5)**	64.7	69.2	66.5	14.3	11.1	22.8	128.8
**5-methoxytryptamine**	65.9	71.7	70.3	71.4	54.6	134.2	ND
**Cortisol**	26.7	33.6	29.1	112.6	108.9	70.3	ND
**Cortisone**	25.9	31.6	28.0	101.1	113.7	71.1	ND
**Melatonin**	93.6	58.8	99.0	104.1	109.8	122.4	ND
**Triiodothyronine**	76.3	83.3	75.4	90.5	105.4	142.8	ND
**Thyroxine**	18.8	25.9	10.3	57.2	18.6	ND	ND
**Sphingosine**	33.7	34.4	34.7	62.6	74.4	46.8	46.5
**PI (19:0)**	64.5	66.9	85.0	83.8	ND	ND	ND
**PE (17:0)**	89.6	103.6	81.7	45.5	ND	ND	ND
**Angiotensin II**	67.6	70.4	71.9	28.4	19.5	74.7	ND
**B. Repeatability (RSD %) of extractions of standard analytes from buffer**							
**Glutamic acid**	25.5	26.4	6.2	N/A	N/A	14.6	41.5
**Tyrosine**	4.6	12.2	5.7	N/A	N/A	9.0	30.8
**Creatinine**	9.0	8.7	5.8	N/A	N/A	N/A	N/A
**Thyrotropin releasing hormone**	4.0	12.6	4.0	42.0	3.0	14.0	11.2
**Pantothenic Acid**	3.9	8.6	1.5	65.0	26.8	13.2	18.9
**Histamine**	7.1	3.3	3.6	N/A	N/A	10.4	27.5
**4-aminobutanoic acid**	5.5	8.6	5.4	N/A	31.0	14.7	N/A
**Adenine**	14.0	20.4	14.1	46.9	N/A	17.1	13.1
**Dopamine**	8.4	11.0	23.4	24.6	25.9	20.8	40.5
**Serotonin**	6.4	2.9	4.1	17.3	22.6	11.7	10.9
**Homovanillic acid**	19.1	6.0	16.1	25.2	52.3	10.2	N/A
**Phenylalanine (d5)**	4.9	2.8	3.0	61.0	21.3	16.1	17.6
**5-methoxytryptamine**	5.5	3.2	4.6	12.3	25.2	3.6	N/A
**Cortisol**	24.6	23.8	27.3	6.8	18.6	29.4	N/A
**Cortisone**	16.9	24.9	18.9	8.7	13.9	27.1	N/A
**Melatonin**	14.4	9.6	12.1	9.0	20.5	16.8	N/A
**Triiodothyronine**	5.2	3.5	2.8	6.1	11.7	6.6	N/A
**Thyroxine**	15.0	56.3	15.6	55.6	18.3	N/A	N/A
**Sphingosine**	1.2	3.0	3.5	3.0	31.9	1.0	0.3
**PI (19:0)**	16.0	10.4	20.4	25.0	N/A	N/A	N/A
**PE (17:0)**	46.8	21	63.7	16.8	N/A	N/A	N/A
**Angiotensin II**	3.9	5.3	3.0	10.5	6.8	2.7	N/A

Supplementary Table 2. Recovery and precision of extractions of standard analytes from buffer. The table displays average recovery calculated from individual replicates (n=6, amount of standard analyte spiked before extraction = 100%) and a relative standard deviation (standard deviation/mean*100%, n=6) of recovered amounts. “ND” stands for “not detected”, “N/A” stands for “not applicable. LogP was obtained from ChemSpider (http://www.chemspider.com/Default.aspx) and represents predicted octanol-water partition coefficients (predicted ACD/LogP, ACD/Labs, Toronto, Canada. For details on the usage and fate of analytes see Supplementary Table 7.

Analytes	Methanol-Ethanol	Methanol	Methanol-MTBE	MTBE	C18	PEP2	IEX
**A. Recovery (% of input) of standard analytes from plasma**							
**Folic acid**	81.5	99.2	44.2	10.8	73.1	139.0	185.7
**Adenine**	91.1	75.3	96.6	13.0	244.0	245.6	ND
**Histamine**	28.8	68.0	46.3	ND	23.2	53.2	129.7
**4-aminobutanoic acid (d6)**	65.6	86.3	74.5	ND	2.4	ND	33.2
**4-aminobutanoic acid**	ND	ND	ND	ND	15.5	ND	ND
**Pantothenic Acid**	110.3	109.3	102.9	7.9	12.6	5.4	95.1
**Tyrosine**	ND	ND	ND	101.6	53.7	18.6	ND
**Homovanillic acid**	98.0	111.6	92.7	8.5	ND	115.6	99.1
**Homovanillic acid (d3)**	105.1	115.0	101.7	8.7	5.0	108.5	96
**Kynurenine**	ND	ND	ND	1.1	152.7	ND	ND
**Melatonin (d4)**	118.0	121.0	119.0	103.9	163.9	158.8	2.2
**Melatonin**	106.1	106.8	104.1	92.6	150.7	157.3	1.5
**5-Methoxytryptamine**	73.1	80.4	66.8	18.8	107.1	109.4	ND
**Cortisol (d4)**	93.4	90.7	80.7	69.4	5.2	2.7	ND
**Cortisol**	98.1	96.4	86.2	73.7	7.3	6.4	ND
**Cortisone**	69.3	74.0	38.3	74.5	2.9	0.4	ND
**Cholic acid**	114.4	123.5	121.4	ND	19.6	138.8	142.9
**Cholic acid (d4)**	114.3	114.6	110.6	ND	12.7	139.7	147.6
**Triiodothyronine**	46.7	56.8	58.8	11.7	90.0	125.3	6.9
**Thyroxin (13C6)**	56.7	77.5	68.5	3.8	84.9	117.0	6.2
**Thyroxine**	60.3	80.8	72.4	4.6	77.1	105.7	5.8
**Sphingosine**	3.9	27.4	10.5	56.6	56.0	75.0	21.4
**Neurotensin**	62.0	68.9	39.3	6.9	143.3	154.7	5.2
**Thyrotropin releasing hormone**	90.7	98.0	82.6	ND	102.9	125.8	134.9
**B. Repeatability (RSD %) of extractions of standard analytes from plasma**							
**Folic acid**	12.8	8.5	15.3	103.0	110.0	46.0	40.0
**Adenine**	8.7	16.3	15.6	69.2	81.2	46.0	N/A
**Histamine**	26.9	13.4	13.7	N/A	60.1	46.0	20.6
**4-aminobutanoic acid (d6)**	8.4	20.8	7.7	N/A	12.1	N/A	14.2
**4-aminobutanoic acid**	N/A	N/A	N/A	N/A	59.0	N/A	N/A
**Pantothenic Acid**	5.4	3.0	3.6	123.8	38.9	42.7	18.0
**Tyrosine**	N/A	N/A	N/A	115.6	61.4	63.8	N/A
**Homovanillic acid**	8.7	5.8	4.0	245.0	N/A	52.1	11.4
**Homovanillic acid (d3)**	5.9	7.0	8.7	235.0	0.5	48.7	12.8
**Kynurenine**	N/A	N/A	N/A	161.9	143.5	N/A	N/A
**Melatonin (d4)**	9.7	6.4	3.1	18.8	6.3	9.5	244.9
**Melatonin**	8.6	4.5	3.4	19.1	6.6	9.9	244.9
**5-methoxytryptamine**	7.0	4.1	5.3	58.2	14.6	22.6	N/A
**Cortisol (d4)**	5.2	5.4	8.9	35.0	91.6	131.4	N/A
**Cortisol**	6.2	7.8	8.8	36.7	58.5	53.8	N/A
**Cortisone**	9.2	7.4	42.5	29.4	93.0	N/A	N/A
**Cholic acid**	11.8	10.7	9.9	N/A	113.3	9.1	40.6
**Cholic acid (d4)**	12.6	9.0	9.8	N/A	110.6	9.6	38.0
**Triiodothyronine**	36.0	23.8	10.4	23.3	10.1	32.4	28.6
**Thyroxin (13C6)**	32.0	27.6	27.1	51.2	13.5	40.9	2.7
**Thyroxine**	33.0	28.8	27.1	45.3	13.7	41.7	1.9
**Sphingosine**	18.2	15.4	20.1	55.6	19.2	81.0	54.3
**Neurotensin**	10.7	7.4	11.0	111.2	43.1	24.7	52.3
**Thyrotropin releasing hormone**	23.8	27.1	22.8	N/Ap	68.4	37.1	10.5

Supplementary Table 3. Recovery and precision of extraction of standard analytes from plasma. The table displays average recovery calculated from individual replicates after subtraction of any endogenous level of metabolite present (n=6, amount of standard analyte spiked before extraction = 100%) and a relative standard deviation (standard deviation/mean*100%, n=6) of recovered amounts. LogP was obtained online from ChemSpider website (http://www.chemspider.com/Default.aspx) and represents predicted octanol-water partition coefficients (predicted ACD/LogP, ACD/Labs, Toronto, Canada). “ND” stands for “not detected”, “N/A” stands for “not applicable”. For details on the usage and fate of analytes see Supplementary Table 8.

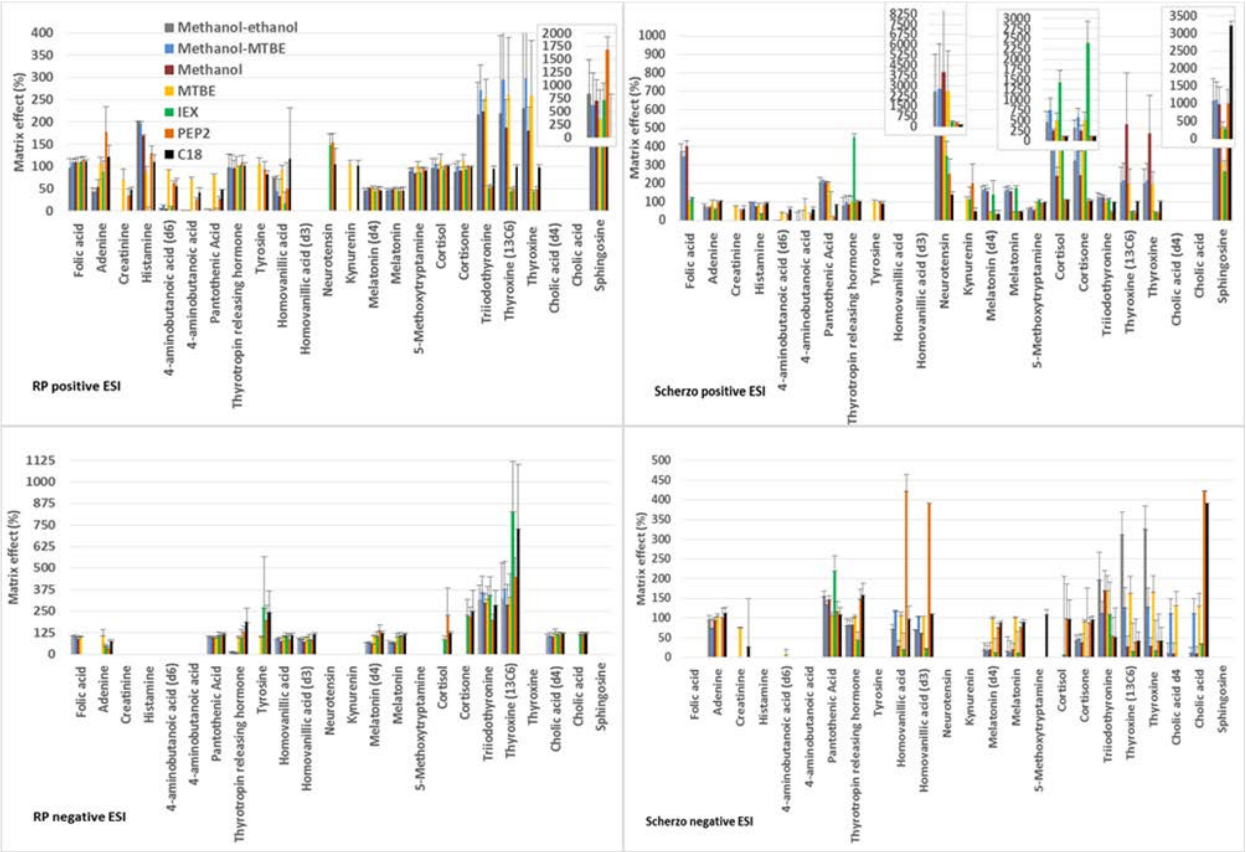

Supplementary Figure 2 Ionization effects observed in human plasma in all LC-MS analysis across all analytes and extraction methods. On Y axis, graph displays area ratios between analytes of the same concentrations in buffer and a matrix calibration points ((area in spiked matrix-area in blank matrix) / (area in buffer-area in blank)*100%)) assuming the area of analyte in the buffer as 100 %. Analytes are displayed along X-axis from left to right according to the increase of their predicted ACD/LogP, ACD Laboratories Toronto, Canada). Legend designating extraction methods on RP positive panel is same for all panels. Error bars represent relative standard deviation of matrix effects between different concentration levels tested. Due to very high matrix effect of some analytes in RP and Scherzo in positive ESI, full scale inserts were placed. Missing bars indicate that matrix effects could not be calculated due to one of three possible events: (i) analyte is not detected in buffer, (ii) analyte is not detected in a particular matrix or (iii) analyte signal was saturated (signal did not increase together with increased concentration at the concentration levels tested). The differences in matrix effect between 4-aminobutanoic acid and its deuterated analog in RP positive ESI (NDM) and Scherzo in positive ESI (methanol) is most likely due to the strong suppressing matrix effect. The differences in matrix effects between cholic acid, thyroxine and their deuterated analogs in RP in negative ESI are most likely due to the error in preparation of calibration points in the correspondent samples where it was not detected. Cortisol (d4) was removed from the graph because it was not detected in neither analysis except RP in positive ESI. See supplementary Table 6 for details.

Extraction method	Buffer				Spiked plasma			
	# of analytes RSD≤20 R≥80%	# of analytes RSD≤30 R≥50 %	Mean R (%)	Mean RSD (%)	# of analytes RSD≤20 R≥80 %	# of ana-lytes RSD≤30 R≥50 %	Mean R (%)	Mean RSD (%)
**Methanol-ethanol**	4	15	65.9	11.9	11	16	80.4	14.3
**Methanol**	5	16	73.1	12.9	11	19	89.6	12.4
**Methanol-MTBE**	5	15	66.2	12.0	10	19	77.1	13.3
**MTBE**	4	10	63.5	25.6	2	3	37.1	85.4
**C18**	4	5	52.9	22.0	5	7	69.8	53.4
**PEP2**	6	11	77.0	13.3	4	6	100.1*	42.5
**IEX**	3	5	71.4	21.2	4	5	69.6	52.2

Supplementary Table 4 Efficiency of extraction methods in buffer and plasma for metabolite standards using the data obtained in RP analysis in positive or negative ESI mode. The first two columns compare the extraction methods using the number of analytes recovered in extractions from buffer and plasma at different thresholds of precision and recovery (R). The number of analytes recovered with RSD less than 20% and R≥80% and can be considered as metabolites where the extraction method provides excellent performance. The metabolites which have RSD less than 30%, and recovery higher than 50% can be considered as metabolites with acceptable performance in semi-quantitative methods such as global metabolomics. The table also shows mean recovery and repeatability (expressed as mean RSD) across all metabolites observed in a given extraction method. The total number of standard analytes detected from standard mixture by at least one extraction method is 22 metabolites for buffer and 24 metabolites for plasma extractions. Supplementary Table 8 summarizes which metabolites were detected/analyzed in only one matrix. *Higher mean recovery of PEP2 is caused by the larger number of analytes with enhanced matrix effect (Table 2) in RP analysis in positive ESI comparative to other extractions.

Metabolite	1-step MTBE extraction	2-step MTBE extraction
Folic acid	8.2 ± 5.6	29.4 ± 14
4-aminobutanoic acid	ND	ND
d6 4-aminobutanoiic acid	ND	ND
Histamine	1.4 ± 0.4	ND
Creatinine	ND	ND
Adenine	2.6 ± 2.8	17.9 ± 4.6
d6-Norepinephrine	ND	ND
Tyrosine	ND	ND
Thyroid releasing hormone	ND	ND
5-Methoxytryptamine	ND	ND
Pantothenic Acid	ND	ND
Cortisone	96.4 ± 7.2	122 ± 13
Cortisol	98.1 ± 7.9	125 ± 10
Melatonin	142 ± 46	144 ± 14
d4 Melatonin	121 ± 41	118 ± 13
Triiodothyronine	13.6 ± 0.2	13.2 ± 3.2
13C6 Thyroxin	8.4 ± 0.1	8.3 ± 0.1
Sphingosine	73.9 ± 14	80.2 ± 5.4
Neurotensin	ND	ND

Supplementary Table 5. Recovery and precision of extractions of standard analytes from plasma in a follow-up experiment where one-step and two-step MTBE extractions were compared. The table displays average recovery calculated from individual replicates (n=6, amount of standard analyte spiked before extraction = 100%) and standard deviation of recovered amounts. “ND” stands for “not detected”,

Analytes	Charge class	Number of methods for which metabolite had ≥80% recovery and ≤20% RSD:		Number of methods without matrix effect			
		in buffer	in plasma	RP +ESI	Scherzo +ESI	RP -ESI	Scherzo - ESI
**Folic acid**	zwit.	NA	2	7	2	4	N/Ap
**Adenine**	pos.	2	2	2	3	1	5
**Histamine**	zwit.	4	0	2	5	N/Ap	4
**4-aminobutanoic acid (d6)**	zwit.	NA	0	1*	0	N/Ap	N/Ap
**4-aminobutanoic acid**	zwit.	3	0	0	0	N/Ap	0
**Pantothenic Acid**	neg.	1	4	0	1	7	3
**Thyrotropin releasing hormone**	zwit.	0	0	7	6	2	4
**Tyrosine**	zwit.	0	0	3	3	1	N/Ap
**Homovanillic acid**	neg.	1	4	2	0	6	3
**Homovanillic acid (d3)**	neg.	NA	4	0	0	6	3
**Neurotensin**	zwit.	NA	0	1	0	N/Ap	N/Ap
**Kynurenine**	zwit.	NA	0	2	2	N/Ap	N/Ap
**Melatonin (d4)**	N	NA	6	0	0	4	2
**Melatonin**	N	4	6	0	0	4	2
**5-Methoxytryptamine**	zwit.	1	2	7	4	N/Ap	1
**Cortisol**	N	2	3	7	2	1	2
**Cortisol (d4)**	N	2	3	7	N/Ap	N/Ap	N/Ap
**Cortisone**	N	2	0	7	2	0	3
**Triiodothyronine**	neg.	4	1	1	3	0	2
**Thyroxine (13C6)**	neg.	NA	1**	1	1	0	0
**Thyroxine**	neg.	0	0**	1	1	N/Ap	0
**Cholic acid**	neg.	NA	4	ND	ND	3***	2
**Cholic acid (d4)**	neg.	NA	4	ND	ND	7	2
**Sphingosine**	zwit.	0	0	0	0	0	0

Supplementary Table *6*. Extraction quality and resistance of standard analytes to matrix effects across LC-MS methods. Analytes ordered top to down accordingly to the LogP oct/water coefficient predicted by ACD laboratories algorithm. "ND" stands for not detected. "N" stands for neutral, "NA" stands not available as standard analyte at the time of extraction, "N/Ap" designates analytes for which matrix effect could not be calculated in any extraction method, "zwit." – for zwitterions, "neg." - for negative, "pos." - for positive considering the presence of either both, acidic or basic groups, respectively. Two columns "Number of methods for which metabolite had ≥80% recovery and ≤20% RSD:" provide the number of extraction methods in which an analyte was detected with RSD less than 20 and recovery higher than 80% in plasma or buffer. The matrix effect section represents the number of extraction methods (total =7) in which matrix effect was not observed for the standard analyte. For the standard analytes marked with asterisk, the matrix effect for isotope analog was just slightly outside 80% limit. Thus, 4-aminobutanoic acid demonstrated 71.6 % matrix effect in MTBE, while its deuterated analog demonstrated 89.4 %, ** recovery of thyroxine was slightly lower (77.1%) then for its deuterated analog (84.9 %). Both of these results lie within normal LC-MS experimental error and are not statistically significantly different. For example, using the above table it can be seen that histamine was recovered quantitatively and reproducibly by four extraction methods from buffer but in no methods from plasma. Histamine also showed no matrix effects in 2 and 5 extraction methods in RP and Scherzo analysis in positive ESI and in 4 extraction methods in Scherzo negative ESI analysis, respectively. *** Cholic acid was not found in any of liquid extractions (4 in total) in RP analysis at negative ESI in contrary to its deuterated analog which was detected in all extractions.

Analytes	RP +ESI							Scherzo +ESI						
	Methanol-ethanol	Methanol-MTBE	Methanol	MTBE	IEX	PEP2	C18	Methanol-ethanol	Methanol-MTBE	Methanol	MTBE	IEX	PEP2	C18
**Folic acid**	95.4	108.2	109.1	106.4	109.9	114.4	111.9	372.8	344.0	400.6	100.5	119.5	***NDM***	***NDM***
**Adenine**	42.4	43.3	52.8	106.2	87.2	176.9	121.9	77.6	62.8	68.9	101.3	57.9	97.6	103.5
**Histamine**	198.9	196.1	168.7	88.4	***SS***	129.9	110.0	99.9	98.9	75.5	82.3	36.4	84.3	93.9
**4-aminobutanoic acid (d6)**	6.2	12.6	4.3	89.4	0.3	62.1	55.2	0.8	1.7	0.7	41.3	***NDM***	30.3	60.1
**4-aminobutanoic acid**	0.7	0.7	0.4	71.2	***NDM***	24.8	40.9	0.2	0.2	***NDM***	75.8	***NDM***	29.1	59.6
**Pantothenic Acid**	***SS***	***SS***	***SS***	77.5	***SS***	26.7	46.6	209.0	213.7	205.9	198.9	***SS***	***SS***	***SS***
**Thyrotropin releasing hormone**	97.5	96.1	95.5	101.0	101.2	106.6	101.5	80.6	97.2	86.3	99.3	450.2	100.3	100.0
**Tyrosine**	***SS***	***SS***	***SS***	105.2	***SS***	92.5	82.9	***NDM***	***NDM***	***NDM***	101.2	***NDM***	99.6	91.0
**Homovanillic acid**	72.7	43.2	32.5	91.8	17.0	50.0	116.6	***SS***	***SS***	***SS***	***SS***	***SS***	***SS***	***SS***
**Homovanillic acid (d3)**	***NDB***	***NDB***	***NDB***	***NDB***	***NDB***	***NDB***	***NDB***	***NDB***	***NDB***	***NDB***	***NDB***	***NDB***	***NDB***	***NDB***
**Neurotensin**	***SS***	***SS***	***SS***	***SS***	148.6	152.7	104.8	2578.3	2750.1	3976.0	2542.4	346.6	250.5	138.7
**Kynurenine**	***SS***	***SS***	***SS***	104.2	***SS***	***SS***	101.7	***NDM***	***NDM***	***NDM***	112.7	115.9	199.8	47.9
**Melatonin (d4)**	45.3	46.1	50.0	50.7	43.8	47.5	45.9	161.7	171.0	157.3	46.6	140.2	35.0	35.3
**Melatonin**	45.2	45.9	47.4	48.3	43.9	45.4	45.7	158.8	168.0	155.7	45.3	172.3	43.8	44.8
**5-Methoxytryptamine**	88.4	92.7	83.8	100.7	86.9	93.9	91.3	57.4	65.7	52.6	92.7	107.3	92.3	97.6
**Cortisol**	95.1	105.1	93.8	111.2	92.1	99.9	99.9	442.4	742.5	240.8	481.7	1433.7	107.3	106.0
**Cortisol (d4)**	93.8	100.6	95.7	108.9	92.5	87.8	94.2	***NDM***	***NDM***	***NDM***	***NDM***	***NDM***	***NDM***	***NDM***
**Cortisone**	87.4	99.7	89.4	111.6	93.7	99.3	98.6	322.6	575.1	244.8	495.4	2391.1	104.7	104.2
**Triiodothyronine**	215.8	270.8	223.4	253.5	51.4	53.4	94.4	123.5	124.1	123.9	102.0	114.3	44.8	96.2
**Thyroxine (^{13}C6)**	219.3	295.1	187.1	261.0	44.3	47.4	98.6	206.3	216.3	519.5	201.7	43.6	40.4	100.9
**Thyroxine**	229.7	296.7	179.2	257.5	42.5	47.2	98.1	202.5	217.2	471.9	193.6	43.2	41.6	101.8
**Cholic acid (d4)**	***NDB***	***NDB***	***NDB***	***NDB***	***NDB***	***NDB***	***NDB***	***NDB***	***NDB***	***NDB***	***NDB***	***NDB***	***NDB***	***NDB***
**Cholic acid**	***NDB***	***NDB***	***NDB***	***NDB***	***NDB***	***NDB***	***NDB***	***NDB***	***NDB***	***NDB***	***NDB***	***NDB***	***NDB***	***NDB***
**Sphingosine**	745.4	825.8	618.1	695.2	352.1	713.4	1677.1	1075.2	1102.0	962.4	313.0	265.1	989.7	3217.0
**Analytes**	**RP -ESI**							**Scherzo -ESI**						
**Folic acid**	96.6	102.5	90.1	102.8	***NDM***	***NDM***	***NDM***	***NDB***	***NDB***	***NDB***	***NDB***	***NDB***	***NDB***	***NDB***
**Adenine**	***NDM***	***NDM***	***NDM***	108.1	47.2	32.9	75.4	95.0	74.4	94.7	104.2	***NDM***	102.5	112.7
**Histamine**	***NDB***	***NDB***	***NDB***	***NDB***	***NDB***	***NDB***	***NDB***	***NDB***	***NDB***	***NDB***	***NDB***	***NDB***	***NDB***	***NDB***
**4-aminobutanoic acid (d6)**	***SS***	***SS***	***SS***	***SS***	***SS***	***SS***	***SS***	***NDM***	***NDM***	***NDM***	8.7	***NDM***	***NDM***	***NDM***
**4-aminobutanoic acid**	***SS***	***SS***	***SS***	***SS***	***SS***	***SS***	***SS***	***NDB***	***NDB***	***NDB***	***NDB***	***NDB***	***NDB***	***NDB***
**Pantothenic Acid**	94.2	95.9	92.0	97.6	108.7	106.4	115.0	155.3	134.5	147.0	105.6	219.7	116.8	108.8
**Thyrotropin releasing hormone**	8.1	10.2	7.4	97.7	96.5	129.6	187.3	80.7	82.3	82.0	103.3	44.3	149.3	158.2
**Tyrosine**	***SS***	***SS***	***SS***	100.9	269.4	197.6	242.5	***NDM***	***NDM***	***NDM***	***NDM***	***NDM***	***NDM***	***NDM***
**Homovanillic acid**	81.7	91.5	75.6	101.2	107.0	85.7	110.3	71.5	118.1	29.1	106.7	20.5	422.1	97.5
**Homovanillic acid (d3)**	87.6	87.6	72.5	101.1	81.2	85.7	116.4	70.6	104.4	61.0	105.3	22.7	392.2	111.2
**Neurotensin**	***NDB***	***NDB***	***NDB***	***NDB***	***NDB***	***NDB***	***NDB***	***NDB***	***NDB***	***NDB***	***NDB***	***NDB***	***NDB***	***NDB***
**Kynurenine**	***NDB***	***NDB***	***NDB***	***NDB***	***NDB***	***NDB***	***NDB***	***NDB***	***NDB***	***NDB***	***NDB***	***NDB***	***NDB***	***NDB***
**Melatonin (d4)**	63.3	67.7	60.2	105.6	101.5	136.4	121.8	19.3	15.5	19.9	99.5	12.9	75.8	88.3
**Melatonin**	63.1	64.8	62.1	100.2	106.5	103.4	116.9	15.9	13.2	19.9	100.6	11.8	76.9	89.2
**5-Methoxytryptamine**	***NDB***	***NDB***	***NDB***	***NDB***	***NDB***	***NDB***	***NDB***	***NDM***	***NDM***	***NDM***	***NDM***	***NDM***	***NDM***	109.3
**Cortisol**	***NDM***	***NDM***	***NDM***	***NDM***	86.5	227.1	123.6	***NDM***	***NDM***	***NDM***	***NDM***	6.1	98.3	97.5
**Cortisol (d4)**	***NDM***	***NDM***	***NDM***	***NDM***	***NDM***	***NDM***	***NDM***	***NDM***	***NDM***	***NDM***	***NDM***	***NDM***	***NDM***	***NDM***
**Cortisone**	***NDM***	***NDM***	***NDM***	***NDM***	225.9	214.8	250.8	42.8	46.8	38.1	90.9	2.8	88.3	95.9
**Triiodothyronine**	315.4	359.2	297.3	320.6	341.6	198.8	286.2	197.0	112.4	170.4	169.9	109.6	54.8	51.5
**Thyroxine (13C6)**	322.5	377.5	286.4	330.7	828.3	447.4	728.4	312.0	127.1	27.4	163.1	16.3	39.4	42.3
**Thyroxine**	***NDM***	***NDB***	***NDB***	***NDB***	***NDB***	***NDB***	***NDB***	326.2	128.0	29.6	167.2	17.8	42.3	42.5
**Cholic acid (d4)**	94.9	105.0	99.0	116.4	114.5	112.1	119.0	9.9	112.3	9.6	131.9	***NDB***	***NDB***	***NDB***
**Cholic acid**	***NDB***	***NDB***	***NDB***	***NDB***	115.0	116.5	121.3	10.8	112.4	10.2	130.8	34.7	422.1	392.2
**Sphingosine**	***NDB***	***NDB***	***NDB***	***NDB***	***NDB***	***NDB***	***NDB***	***NDB***	***NDB***	***NDB***	***NDB***	***NDB***	***NDB***	***NDB***

Supplementary Table 7 Summary of matrix effects observed for all metabolites across all extraction methods and LC-MS analyses. Not all analytes were successfully detected in buffer (NDB) or in an extract (NDM) which made the calculation of matrix effect impossible as specified. Analytes which did not respond to increased concentrations of spiked standards or which exhibited negative signal response were considered to be suppressed or saturated (SS) and were not used for calculation of matrix effects.

Analytes	Detection in extractions from buffer	Detection in extractions from plasma
**4-aminobutanoic acid (d6)**	NA	+
**Angiotensin II**	+	Replaced by Neurotensin
**Cholic acid**	NA	Added to supplement analysis in negative ESI
**Cholic acid (d4)**	NA	Added to supplement analysis in negative ESI
**Cortisol (d4)**	NA	+
**Creatinine**	+	Excluded (too high endogenous concentration)
**Dopamine**	+	ND
**Dopamine (d4)**	NA	ND
**Folic acid**	ND	+
**Glutamic acid**	+	Excluded (too high endogenous concentration)
**Homovanillic acid (d3)**	NA	Added to supplement analysis in negative ESI
**Kynurenine**	NA	+
**Melatonin (d4)**	NA	+
**Neurotensin**	NA	Added to represent large peptides
**Phenylalanine (d5)**	+	Used as internal reference
**PI (18:3/22:4)**	+	ND
**PE (17:0/17/0)**	+	ND
**Serotonin**	+	ND
**Thyroxine (13C6)**	NA	+

Supplementary Table 8 Usage and fate of standard analytes in targeted quantitation experiments in buffer and plasma. NA- not available at the time of analysis; ND – not detected. Inability to detect PE and PI phospholipids in plasma and PC in buffer and plasma is most likely is due to a high suppression effect at the end of chromatogram caused by LC-MS methodology which was not optimized for lipid metabolites.
